# Supplementary material for: Easy Expression and Purification of Fluorescent N-Terminal BCL11B CCHC Zinc Finger Domain
Source: Molecules. 2021 Dec 14;26(24):7576. doi: 10.3390/molecules26247576 (PMC8708588; doi:10.3390/molecules26247576)
Supplement: Supplementary file 1 [file molecules-26-07576-s001.zip › molecules-1491503-supplementary.pdf]

Supplementary Information

Easy Expression and Purification of Fluorescent N-Terminal BCL11B CCHC Zinc Finger Domain

Anne Susemihl [1,2], Felix Nagel [1], Piotr Grabarczyk [2], Christian A. Schmidt [2], Mihaela Delcea [1*]

[1] Department of Biophysical Chemistry, Institute of Biochemistry, University of Greifswald, 17489 Greifswald, Germany; anne.susemihl@uni-greifswald.de (A.S.); felix.nagel@uni-greifswald.de (F.N.)
[2] Department of Hematology and Oncology, Internal Medicine C, University of Greifswald, 17489 Greifswald, Germany; piotr.grabarczyk@med.uni-greifswald.de (P.G.); christian.schmidt@med.uni-greifswald.de (C.A.S.)
* Correspondence: delceam@uni-greifswald.de (M.D.)

```
HHHHHHMVSK GEELFGGIVP ILVELEGDVN GHRFSVSGEG EGDATYGKLT
LKFICTTGKL PVPWPTLVTT LTWGVQCFSR YPDHMKQHDF FKSVMPEGYV
QERTIFFKDD GNYKTRAEVK FEGDTLVNRI ELKGIDFKED GNILGHKLEY
NYISHNVYIT ADKQKNGIKA HFKARHNITD GSVQLADHYQ QNTPIGDGPV
ILPDNHYLST QSKLSKDPNE KRDHMVLLEF VTAAGITHGM DELYKSGGGG
SGGGGSGGGG SMENLYFQGL ESGLGLMVGG PDPDLLTCGQ CQMNFPLGDI
LVFIEHKRKQ CGGSLGACYD KALD
```

Figure S1. Amino acid sequence of CyPet-BCL11B$_{42\text{-}94}$. Specific sequences are underlined: Grey – His$_6$-tag. Blue – CyPet protein tag. Violet – 3xGGGGS linker. Green – TEV cleaving site. Pink – BCL11B$_{42\text{-}94}$ domain. The digest of CyPet-BCL11B$_{42\text{-}94}$ using TEV protease results in untagged BCL11B$_{42\text{-}94}$ with three additional amino acids at the N-terminus of the protein.

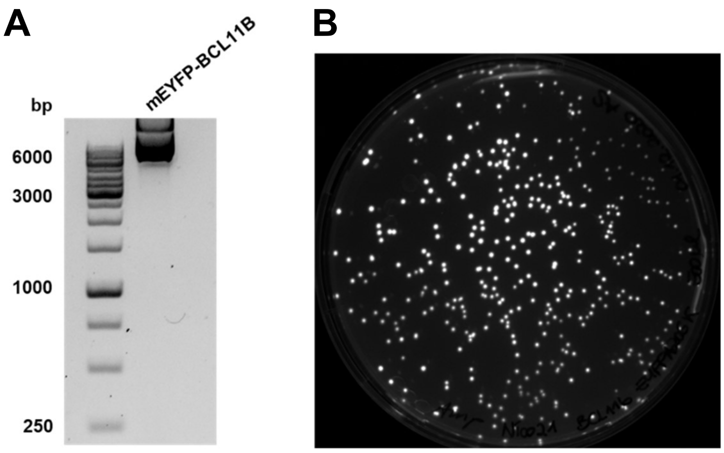

Figure S2. (**A**) Successful mutagenesis of EYFP-BCL11B$_{42\text{-}94}$ A206K mutation to obtain monomeric EYFP (mEYFP). The amplified PCR product at 6620 bp is shown on an 1 % agarose gel and the mutation was verified by sequencing. (**B**) Fluorescent positive mEYFP-BCL11B$_{42\text{-}94}$ colonies that were picked for sequencing and large-scale expression.

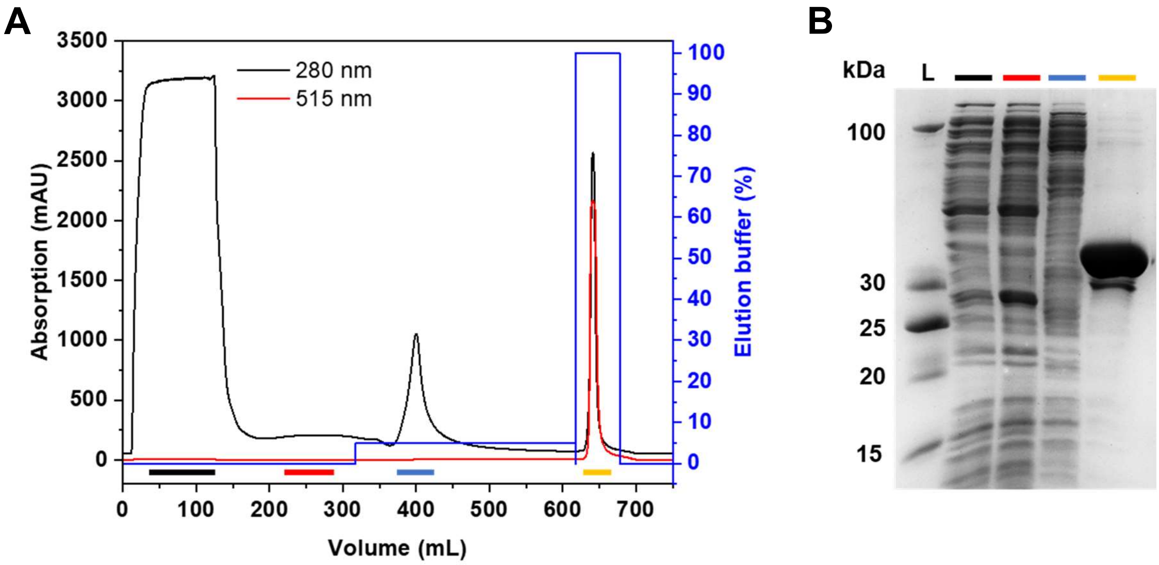

Figure S3. Purification of mEYFP-BCL11B$_{42\text{-}94}$. (**A**) Chromatogram of mEYFP-BCL11B$_{42\text{-}94}$ purification using IMAC. (**B**) Corresponding SDS-PAGE showing collected fractions. The flow-through (black) shows *E. coli* proteins that do not bind to the column. The wash (red) contains loosely bound proteins. The wash step with 5% elution buffer (blue) is crucial to obtain the highly pure fluorescent zinc finger domain (yellow). The small band right below the protein of interest is a small part of only mEYFP-tag without the BCL11B$_{42\text{-}94}$ part.

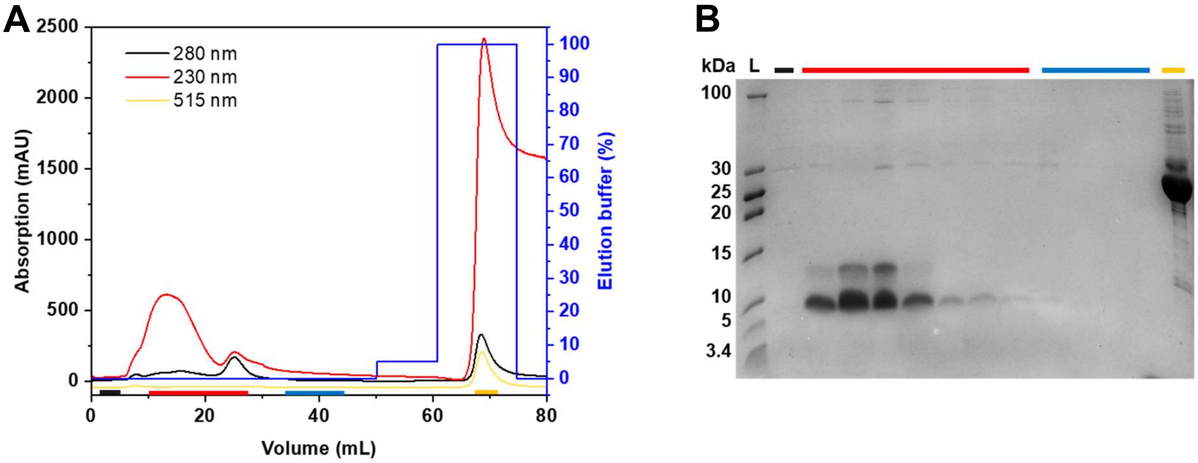

Figure S4. Purification of untagged BCL11B$_{42\text{-}94}$ after TEV digest of mEYFP-BCL11B$_{42\text{-}94}$. (**A**) IMAC chromatogram of the TEV digested mEYFP-BCL11B$_{42\text{-}94}$. (**B**) Corresponding tricine SDS-PAGE of the collected fractions. Black fractions show the first fractions of the flow-through. Red fractions contain the BCL11B$_{42\text{-}94}$ zinc finger domain in high purity and concentration. The wash is shown in blue, with no visible protein content. The mEYFP-tag elutes with 100% elution buffer and is present in the yellow fraction. The second band around 12 kDa marks the dimer of BCL11B$_{42\text{-}94}$.

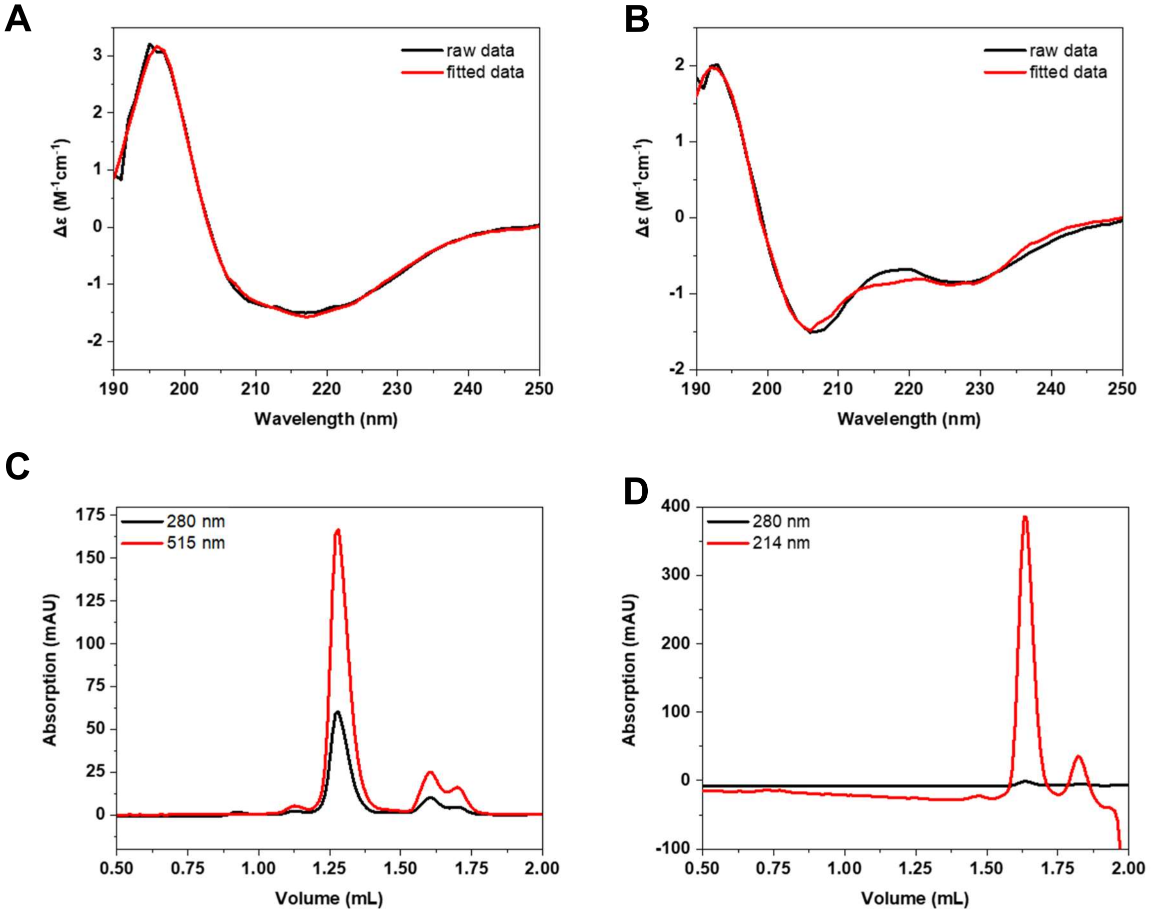

Figure S5. (**A**) CD spectrum of mEYFP-BCL11B$_{42-94}$ with raw data shown in black and the corresponding fit for deconvolution shown in red. (**B**) CD spectrum of untagged BCL11B$_{42-94}$ obtained after digesting mEYFP$_{42-94}$ using TEV protease. Size exclusion chromatography of (**C**) mEYFP-BCL11B$_{42-94}$ and (**C**) untagged BCL11B$_{42-94}$ after TEV digest of mEYFP-BCL11B$_{42-94}$. Elution volumes of 1.35 mL correspond to complexes of ~150 kDa, 1.6 mL to ~40 kDa (monomer) and 1.7 mL to ~30 kDa (mEYFP-tag).

Table S1. Secondary structure content of BCL11B$_{42-94}$ after TEV digest of mEYFP-BCL11B$_{42-94}$. Deconvolution was carried out using BeStSel.com and secondary structure content is shown in percentage [%].

**Secondary Structure**	**BCL11B$_{42-94}$**
α-Helix	9.4
Antiparallel β-sheet	27.8
Parallel β-sheet	0.0
β-Turn	16.1
Others	46.6

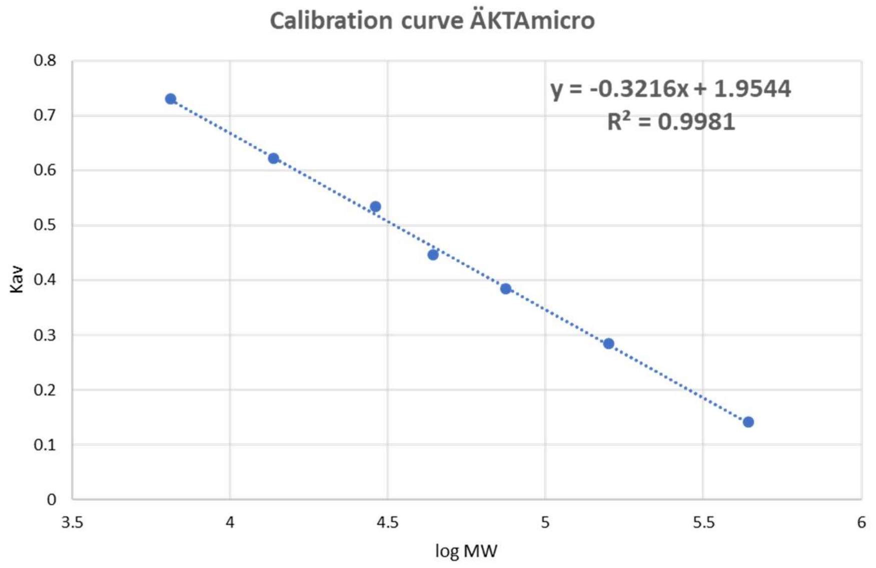

Figure S6. Calibration curve for a Superdex 200 Increase 3.2/300 column used for determination of molecular weight in dependence from elution volume. The used proteins and their molecular weight are shown in Table S1.

Table S2. Proteins and their molecular weight (MW) used for calibration of a Superdex 200 Increase 3.2/300 column. Determination of Kav was carried out according to instructions by Cytiva.

**Protein**	**MW [Da]**	**log MW**	**K_{av}**	**Elution Volume [mL]**
Ferritin	440000	5.64345268	0.14189189	1.13
Aldolase	158000	5.19865709	0.28378378	1.34
Conalbumin	75000	4.87506126	0.38513514	1.49
Ovalbumin	44000	4.64345268	0.44594595	1.58
Carbonic Anhydrase	29000	4.462398	0.53378378	1.71
Ribunuclease A	13700	4.13672057	0.62162162	1.84
Aprotinin	6500	3.81291336	0.72972973	2
